# Overview of the Pathophysiological Implications of Organotins on the Endocrine System

**DOI:** 10.3389/fendo.2018.00101

**Published:** 2018-03-16

**Authors:** Vinicius Bermond Marques, Rodrigo Alves Faria, Leonardo Dos Santos

**Affiliations:** ^1^Department of Physiological Sciences, Federal University of Espirito Santo, Vitoria, Brazil; ^2^Pitagoras College, Guarapari, Brazil; ^3^Department of Health Sciences, Federal University of Espirito Santo, São Mateus, Brazil

**Keywords:** tributyltin, triphenyltin, impossex, endocrine disruptor, obesogen, metabolic disrupting chemicals, hypothalamus–pituitary axis

## Abstract

Organotins (OTs) are pollutants that are used widely by industry as disinfectants, pesticides, and most frequently as biocides in antifouling paints. This mini-review presents the main evidences from the literature about morphophysiological changes induced by OTs in the mammal endocrine system, focusing on the metabolism and reproductive control. Similar to other toxic compounds, the main effects with potential health risks to humans and experimental animals are not only related to dose and time of exposure but also to age, gender, and tissue/cell exposed. Regarding the underlying mechanisms, current literature indicates that OTs can directly damage endocrine glands, as well as interfere with neurohormonal control of endocrine function (i.e., in the hypothalamic–pituitary axis), altering hormone synthesis and/or bioavailability or activity of hormone receptors in the target cells. Importantly, OTs induces biochemical and morphological changes in gonads, abnormal steroidogenesis, both associated with reproductive dysfunctions such as irregular estrous cyclicity in female or spermatogenic disorders in male animals. Additionally, due to their role on endocrine systems predisposing to obesity, OTs are also included in the metabolism disrupting chemical hypothesis, either by central (e.g., accurate nucleus and lateral hypothalamus) or peripheral (e.g., adipose tissue) mechanisms. Thus, OTs should be indeed considered a major endocrine disruptor, being indispensable to understand the main toxic effects on the different tissues and its causative role for endocrine, metabolic, and reproductive dysfunctions observed.

## Introduction

Organotins (OTs) belong to a class of pollutants described as organometallic used for various industrial purposes as disinfectants of water for industrial refrigeration, pesticides, biocides in antifouling paints, and wood preservatives (1–11).

Actually, tin-based compounds are known since the bronze age in the production of different metal alloys (12). However, the industrial use was consolidated only around 1940 as an efficient chemical stabilizer for plastic manufacture (5). Afterward, the biocidal effect of OTs was discovered and thus became intensively employed in a number of other commercial purposes. In this context, it is worth noting the use as an active principle of antifouling paints for boats and ships, reaching the apex in the 1990s, when about 80% of the boats worldwide used OT-based products (1, 3, 4).

Tin usually binds to non-polar radicals resulting in hydrophobic compounds; and due to their physicochemical properties, OTs are easily absorbed along the food chain. The effects depend greatly on the number and nature of radicals bound to the tin atom, being the tri-substituted (triorganostannic) forms, such as tributyltin (TBT) and triphenyltin (TPT) the most toxic. Fortunately, TBT is degraded in the environment to dibutyltin and then to monobutyltin (5, 12–16).

The TBT toxicology has become a major concern for the scientific community since the 1970s when toxic effects were discovered in different animal models, including mammals. As a result, researches were driven to better understand the actual impact of OT pollution for health and environmental risk (17, 18). These compounds can be easily assimilated by living organisms; in marine environment, for example, OTs are incorporated into soil and organic surface sediments such as phytoplankton, being absorbed by animals and plants of aquatic ecosystems (5, 19).

**Figure 1:**
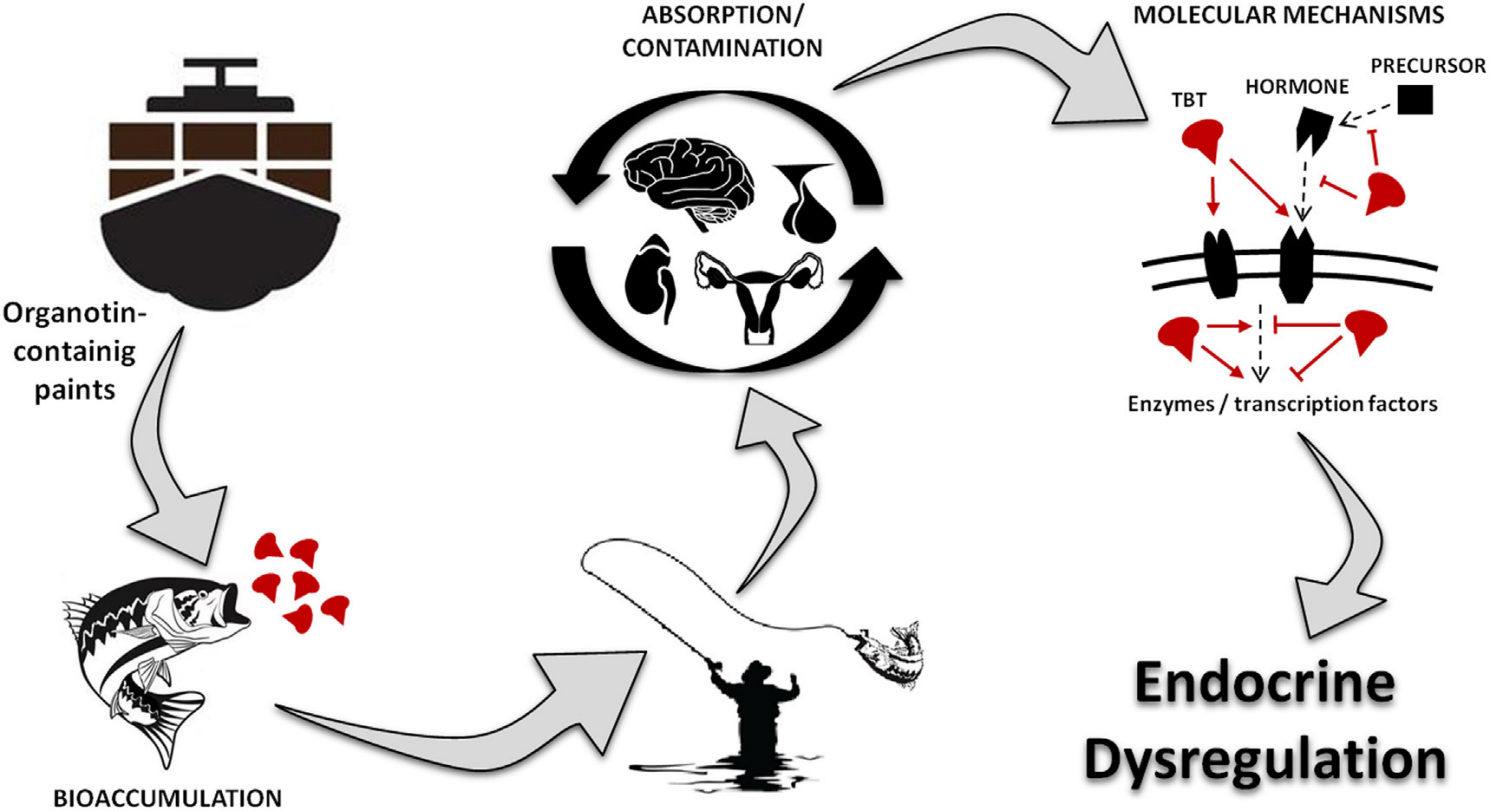
Visual abstract showing the main route of exposure to organotins for humans, underlying toxic mechanism and the potential consequences for the endocrine system. TBT, tributyltin.

Figure 1 represents a visual summary of the main route of exposure to OTs for humans and the potential consequences for the endocrine system. Studies have shown that OTs cause several damages, including genetic, hepatic, renal, adrenal, neural, and immune toxicity (20–24). More importantly, recent reports indicate that TBT is a highly persistent chemical in the environment and food chain, being considered one of the largest existing endocrine disruptor with consequences to different hormonal functions (3, 20, 21, 25–27).

**Figure 2:**
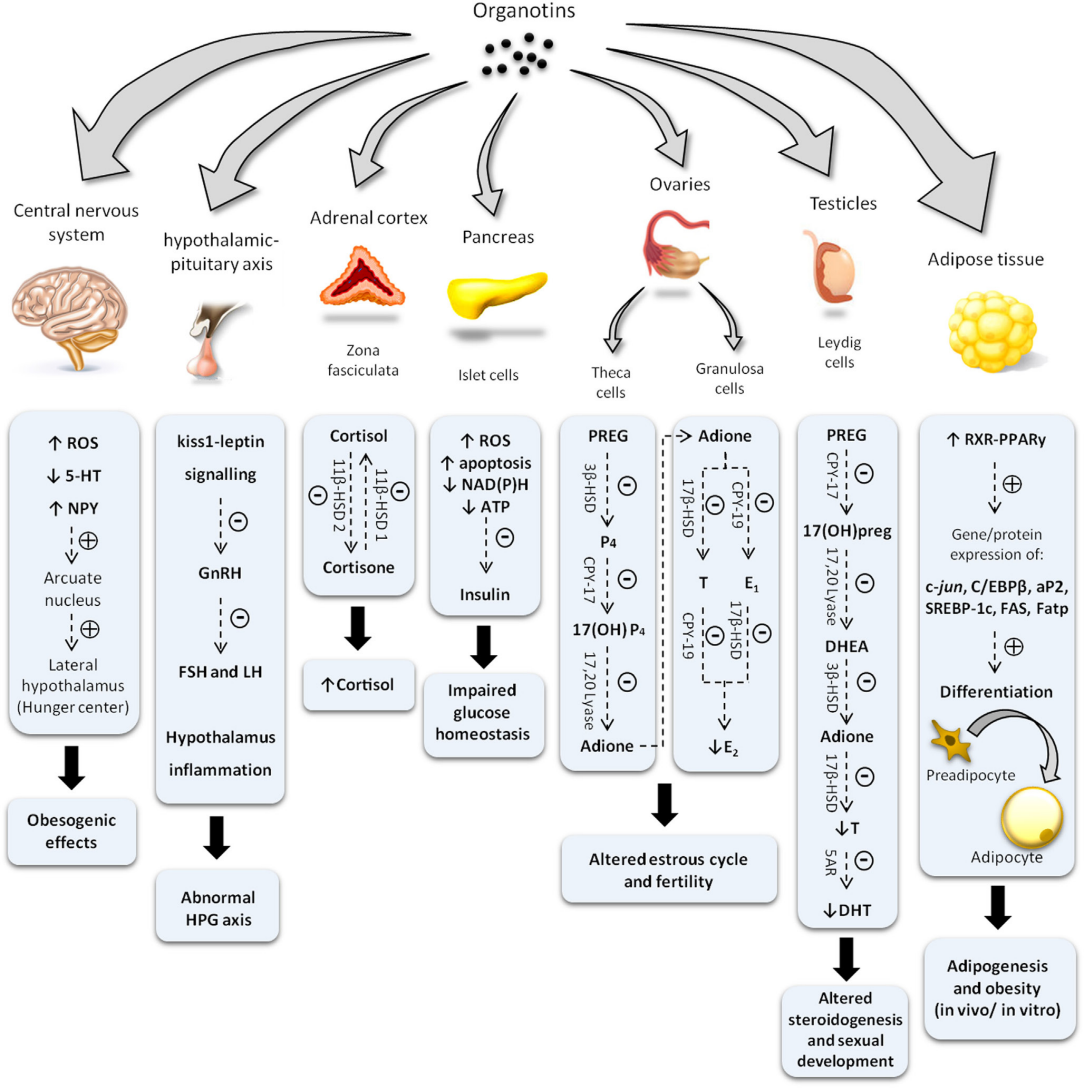
Diagram illustrating changes in endocrine system induced by organotin and major mechanisms involved. Symbols (−) or (+) indicate inhibitory and stimulatory effects, respectively. ROS, reactive oxygen species; 5-HT, serotonine; NPY, neuropeptide Y; GnRH, gonadotropin-releasing hormone; FSH, follicle-stimulating hormone; LH, luteinizing hormone; HPG, hypothalamus–pituitary–gonad; ATP, adenosine triphosphate; NAD(P)H, nicotinamide adenine dinucleotide phosphate; 11β-HSD, 11 β-hydroxysteroid dehydrogenase; PREG, pregnenolone; 3β-HSD, 3β-hydroxysteroid dehydrogenase; P4, progesterone; E1, estrone; E2, 17-β estradiol; T, testosterone; CPY-17, 17α-hydroxylase; Adione, androstenedione; CPY-19, aromatase; 17(OH)preg, 17(OH) pregnenolone; DHEA, dehydroepiandrosterone; 5AR, 5α-reductase; DHT, dihydrotestosterone; C/EBPβ, CCAAT/enhancer binding proteins; SREBP-1c, sterol regulatory element-binding protein 1c; aP2, adipocyte-protein 2; FAS, fatty acid synthase; Fatp, fatty acid transport proteins.

As illustrated at Figure 2 and described in this mini-review, OTs are capable of altering the endocrine physiology at numerous levels: changing the pattern of hormone regulation, production, mechanisms of action or hormone elimination, and mimicking or blocking hormonal action (27–31). In this way, it is not possible to point out an exact toxic effect, whether acting directly on the endocrine glands, compromising hormonal receptors at the target cells, or both. Among all, one of the most iconic effects was noted in contaminated shellfish. These organisms undergo a phenomenon denominated “imposex,” that is the superposition of male genital organs in female individuals (32, 33). Notwithstanding, this endocrine disruptor has been proven as able to reduce circulating estrogen levels and cause morphophysiological damages also in reproductive organs of vertebrates, including mammals (5, 20, 21, 26, 34).

### Neuroendocrine Changes in Mammals Induced by Organotin: A Focus on the Metabolism and the Reproductive Function

In mammals, OTs administered at different doses induce morphological and functional changes in several tissues involved in the control of endocrine function and metabolism, such as the, hypothalamus, pituitary, pancreas, gonads, adipose tissue, adrenal, and thyroid glands (35–38).

The role of environmental pollutants such as OTs on the endocrine system also supports the metabolism disrupting chemical hypothesis (formerly termed “obesogen hypothesis”), which postulates that several environmental toxic chemicals, by altering the endocrine function, can induce metabolic changes related to obesity, impaired glucose metabolism, and dyslipidemia (39, 40). These endocrine and metabolic disorders caused by OTs, particularly obesity, may occur by central and peripheral mechanisms (41, 42). In fact, there are evidences that morphofunctional changes in both fatty tissues and central nervous system may contribute to the deleterious effects of these compounds related to obesity and metabolic syndrome complications (3, 20, 42–47).

In the central nervous system, OTs promote important neurotoxic effects with changes on behavior, metabolism, and neuroendocrine control (48–51). Experimental studies have found decreased levels of dopamine, noradrenaline, and serotonin in mice brains (48), reduced neuron counting and increased glutamate-induced calcium permeability in neuronal membrane from rats (49), and an increase in reactive oxygen-derived species and oxidative damage associated with reduced antioxidant reserves in the nervous system exposed to TBT (50, 51). TBT also exerts its toxicity on other regions of the nervous system, as it is shown by disruption of the rat hypothalamic–pituitary–adrenal axis (22). In addition, the effects of OTs exposure on the brain are not restricted to general neurotoxic effects, but also to changes in neurohormonal control of metabolism and food intake. TBT administered acutely in mice activates the arcuate nucleus and the hunger center of the hypothalamus (46), and in rats increases neuropeptide Y (NPY) expression in the brain, in association with increased body weight, fat mass, and food intake (47). In addition, mice chronically exposed to low doses of TBT exhibited increased food efficiency and reduced leptin circulating levels associated with changes on the leptin–NPY–NPY–Y1 receptor axis in the hypothalamus (52). In this regard, it is well known the importance of leptin modulating the expression of NPY in the hypothalamus and, thus, the food intake. In fact, OT-induced changes on the leptin-NPY axis are associated with obesity due to increased food intake and decreased energy expenditure (53).

In relation to peripheral mechanisms involved in the obesogenic effect, it is well described the association between tin-based compounds and adipogenesis, through signaling between retinoid X receptor (RXR) and peroxisome proliferator-activator receptor gamma (PPARγ) (20, 25, 54–57). There are evidences that TBT increases adipocyte markers expression, lipid accumulation and glucose uptake in preadipocytes (36, 58, 59), and induces a differentiation to adipocytes by RXR/PPARγ activation (42–45). Notwithstanding, it is know that PPARγ plays also an orexigenic role, attributed to its central effects especially in both the NPY and agouti-related protein at the arcuate hypothalamic nucleus (60, 61). Since PPARγ is one of the peripheral targets of TBT, it is possible to speculate that this receptor may be also involved in the central effects related to OTs.

It is important to mention that there is a clear relationship between thyroid function and obesity, with changes in both thyroid-stimulating hormone and thyroxine (T_4_) associated with changes in body weight and fat mass (62). In spite of few studies investigating the OT effects on the hypothalamic–pituitary–thyroid axis, there is a body of evidences indicating that TBT can also be considered a thyroid disruptor, thus contributing to the development of metabolic disorders and obesity (38, 63–65).

Furthermore, pancreas is a key target organ of metabolic disrupter chemicals not only for controlling glucose metabolism but also for modulating digestion (i.e., releasing digestive enzymes) and food intake (e.g., insulin can modulate hypothalamic center of hunger). In fact, in addition to the endocrine and metabolic changes described above, it is known that OTs affect both exocrine (66) and endocrine functions of the pancreas (20, 67–70). Regarding the later, it is proposed that the impairment in the glucose homeostasis occurs probably due to the ability of OTs to reduce insulin secretion and/or signaling, through inhibition of β cell proliferation, increased apoptosis, and decreased production of NAD(P)H and adenosine triphosphate (ATP) in pancreatic islet cells, associated with local oxidative stress (20, 67–70).

It is well known that the impacts on neuroendocrine control caused by OTs also interfere with reproductive endocrine function: TBT exposure was accountable for morphological changes, such as weight loss of the male and female reproductive organs (30, 31, 71) and abnormal steroidogenesis on gametes (72), as well as reproductive dysfunctions such as changes in ovary morphology and abnormal estrous cyclicity (26, 36, 73–75). Interestingly, Podratz et al. (26) demonstrated that the ingestion of seafood homogenate with imposex indeed provoke important alterations in the rat reproductive organs, strengthening the hypothesis that the ingestion of contaminated shellfish is an important source of exposure to OTs (26).

In female rats, TBT oral administration not only induced estrous cycle and ovary morphological abnormalities (i.e., increased apoptotic cells in the corpus luteum and granulosa cells and increased cystic follicles) but also reduced 17β-estradiol and elevate progesterone serum levels (74, 75). Moreover, studies demonstrate that, depending on the dose, TBT can activate estrogen receptors (ER) *in vivo* and *in vitro* having estrogenic and adipogenic activities (3); reduce ER function on metabolic and reproductive controls (20, 26); or even change ER expression in different sites of the hypothalamus–pituitary–gonadal (HPG) axis (75). Actually, the effects of TBT on the gonad function may be, at least in part, due to changes on the HPG axis. Recent studies reported significant alterations in pituitary and hypothalamic morphophysiology and reduced GnRH expression that was related to an impaired Kisspeptin/leptin signaling (22, 75).

Similarly, male adult rats exposed to TBT exhibit varied endocrine damages, including effects on the reproductive endocrine system (30, 71, 76–80). Using different doses, studies with rodents evidenced changes in gonad weight (71, 80), reduced level of luteinizing hormone and testosterone, and spermatogenic disorders associated with reduced Leydig (30, 79) and Sertoli cells (81).

In view of the changes described in the HPG axis and gonads from both genders, an impaired reproductive function should be expected. In fact, several studies have shown that exposure to OTs, in a dose-dependent manner, reduces fertility and embryonic implantation and causes teratogenesis (75, 82–87). Moreover, when administered to pregnant females, TBT-induced weight loss in mothers and their offspring, as well as growth retardation (76, 88). The *in utero* exposure to TBT also leads to an impaired sexual development by affecting germ cells, which may lead to permanent damage in the adult gonads (77). However, there are evidences that perinatal exposure to OTs in rodents affects differentially male and female pups: while male postnatal development was severely affected with decreased weight of reproductive organs, testosterone level and sperm motility, suggesting that impacts may persist throughout adulthood; female pups exhibited more discreet changes such as initiation of estrous cycling and opening of the vagina occurring at an earlier stage. If considering that the enzyme cytochrome P450 aromatase (P450arom) activity is differentially influenced by OTs in male and female organisms, these studies strengthen the hypothesis of the greater susceptibility of males in the pre- and postnatal periods (72, 89–92).

Taking together, the current literature presents strong evidences of OT-induced endocrine dysfunctions, including significant differences between genders following chronic exposure. This is probably due to the ability of OTs causing not only general toxic effects but also specific molecular and cellular changes, thus altering cell signaling in different ways according to the physiology of each organism exposed.

### Major Mechanisms on Metabolic and Endocrine Disrupting Induced by OTs

It is well known that OTs compounds induce their metabolic and endocrine-disrupting effects through interactions with transcriptional regulators such as nuclear and steroid receptors (42). Thus, OTs may affect different nuclear receptor signaling pathways inducing a variety of morphophysiological effects as reviewed herein. For example, as discussed above, OTs exerts obesogenic effect not only by stimulating adipogenesis as agonists of the PPARγ but also by central effects potentially *via* RXR/PPARγ signaling. Moreover, an equally well-described mechanism is to modulate the expression and/or activity of key enzymes for a number of biochemical processes involved in metabolism and endocrine function.

The synthesis of steroid hormones, for example, involves a number of steps catalyzed by enzymatic reactions that are potential targets for OTs including: (1) cholesterol metabolism, (2) chemical enzymatic conversions, and (3) trafficking of molecules between mitochondria and endoplasmic reticulum (93). Thus, OTs may induce biochemical and endocrine disorders due to this capability of up- or downregulate key enzymes of steroidogenesis (74, 75, 94–96). Studies have described a relationship between endocrine dysfunction induced by OTs and their effects on the enzyme P450arom, which converts androgens into estrogen (94, 96, 97). TBT is reported as a competitive inhibitor of P450arom by reducing its affinity for androstenedione, although this inhibitory effect depends on the exposed tissue, concentration, and time of exposure (94, 96, 97). Conversely, Nakanishi et al. (98) demonstrated that TBT and TPT can increase P450arom activity in a dose- and time-dependent manner in human placental choriocarcinoma cells (98). In addition, in male rats OTs increase P450arom activity and reduce testosterone levels, opposite effects to that found in females (30, 78, 89, 90). Thus, the effects of OTs on the enzymes activity vary not only with tissue or exposed cells, dose, and time of exposure but also according to gender, especially in the case of enzymes related to sex hormones. In animal studies are described an OTs-mediated inhibition of 17-hydroxylase, 3-β-HSD, and 17β-HSD, thus suppressing testosterone biosynthesis (99, 100). Furthermore, studies in human blood and tissue samples also evidenced an inhibitory effect on 5α-reductase 1 and 2, P450arom, 3β-HSD 2, 17β-HSD 1, 11β-HSD1, and 3 (101–103). In a molecular level, Lo et al. (101) suggest there is an interaction of OTs with critical cysteine residues of enzymes leading to disturbances in the steroid hormone levels (101).

It is worth noting that in addition to interaction with nuclear and steroid receptors or specific changes on enzymes involved with steroidogenesis as cited above, the endocrine dysfunction due to OTs exposure can be mediated also by general toxic effects, such as increased oxidative stress and damages to mitochondrial function and subsequent responses to cellular stress (104–107). In this regard, the inhibition of ATP synthesis evidenced by studies with OTs exposure could thereby trigger similar biochemical and/or endocrine dysfunctions (108–111).

Finally, based on studies with cells, tissues and living organisms including mammals exposed to OTs, there are strong evidences of the potential toxicity predisposing to metabolic syndrome complications and endocrine-reproductive disorders, due to changes in all components along the hypothalamus–pituitary axis and peripheral tissues. Notwithstanding, there are changes in different sites including adipose tissue, endocrine glands, neurohormonal, and metabolic control centers, which together can justify the role of OTs as an endocrine and metabolism disruptor in mammals.

## Author Contributions

LS and VM idealized the general structure of the text, RF and VM did the literature review, LS, RF, and VM wrote the text, idealized and designed the figures. LS did the final revision of the text.

## Conflict of Interest Statement

The authors declare that the research was conducted in the absence of any commercial or financial relationships that could be construed as a potential conflict of interest.
